# Genes related to apoptosis predict necrosis of the liver as a phenotype observed in rats exposed to a compendium of hepatotoxicants

**DOI:** 10.1186/1471-2164-9-288

**Published:** 2008-06-16

**Authors:** Lingkang Huang, Alexandra N Heinloth, Zhao-Bang Zeng, Richard S Paules, Pierre R Bushel

**Affiliations:** 1 Biostatistics Branch, National Institute of Environmental Health Sciences, Research Triangle Park, North Carolina, USA; 2 Environmental Stress and Cancer Group, National Institute of Environmental Health Sciences, Research Triangle Park, North Carolina, USA; 3 Microarray Group, National Institute of Environmental Health Sciences, Research Triangle Park, North Carolina, USA; 4 Bioinformatics Program, North Carolina State University, Raleigh, North Carolina, USA; 5 GlaxoSmithKline, Research Triangle Park, North Carolina 27709, USA

## Abstract

**Background:**

Some of the biochemical events that lead to necrosis of the liver are well-known. However, the pathogenesis of necrosis of the liver from exposure to hepatotoxicants is a complex biological response to the injury. We hypothesize that gene expression profiles can serve as a signature to predict the level of necrosis elicited by acute exposure of rats to a variety of hepatotoxicants and postulate that the expression profiles of the predictor genes in the signature can provide insight to some of the biological processes and molecular pathways that may be involved in the manifestation of necrosis of the rat liver.

**Results:**

Rats were treated individually with one of seven known hepatotoxicants and were analyzed for gene expression by microarray. Liver samples were grouped by the level of necrosis exhibited in the tissue. Analysis of significantly differentially expressed genes between adjacent necrosis levels revealed that inflammation follows programmed cell death in response to the agents. Using a Random Forest classifier with feature selection, 21 informative genes were identified which achieved 90%, 80% and 60% prediction accuracies of necrosis against independent test data derived from the livers of rats exposed to acetaminophen, carbon tetrachloride, and allyl alcohol, respectively. Pathway and gene network analyses of the genes in the signature revealed several gene interactions suggestive of apoptosis as a process possibly involved in the manifestation of necrosis of the liver from exposure to the hepatotoxicants. Cytotoxic effects of TNF-α, as well as transcriptional regulation by JUN and TP53, and apoptosis-related genes possibly lead to necrosis.

**Conclusion:**

The data analysis, gene selection and prediction approaches permitted grouping of the classes of rat liver samples exhibiting necrosis to improve the accuracy of predicting the level of necrosis as a phenotypic end-point observed from the exposure. The strategy, along with pathway analysis and gene network reconstruction, led to the identification of 1) expression profiles of genes as a signature of necrosis and 2) perturbed regulatory processes that exhibited biological relevance to the manifestation of necrosis from exposure of rat livers to the compendium of hepatotoxicants.

## Background

Hepatotoxicity is one of the most commonly observed adverse effects in response to many environmental and toxic exposures and is of major concern in the drug development industry [1]. The liver's response to insults depends on the properties of the stressor, the dose received and if the exposure is acute or chronic. Examples of injury or damage are fatty liver, necrosis, cholestasis, cirrhosis and cancer. Traditionally, the detection of a stressor's toxicity relies on the evaluation of serum enzyme levels that are indicators of tissue damage [2]. For instance, elevations of ALT and AST are indicative of liver damage [3]. They are associated with inflammation and\or injury to hepatocytes. Necrosis of the liver usually results in hepatocellular plasma membrane leakage of AST and ALT into the bloodstream. However, although the elevated levels of these serum enzymes are indicators of hepatocellular damage, they are poor prognosticators for the severity of the liver injury or acute liver failure. The ability to predict necrosis at the molecular level, the extent (level) of damage and the source of the insult is currently a challenge using classical toxicologic assays, parameters and biomarkers.

Microarray analysis has evolved as a reliable technology to survey the expression of genes across an entire genome [4]. Several efforts have shown that gene expression signatures can be "anchored" to the phenotype of biological samples [5] and even characterize the genetic variability in individuals [6,7]. The possibilities of phenotypic anchoring are promising and just beginning to take form in investigations from toxicogenomics and risk assessment to pharmacogenomics and personalized medicine. Ultimately, the success of these efforts relies on the identification of genes and gene products that are considered biomarkers of toxicity or candidates of susceptibility to health conditions. Recently, Bushel et al. [8] have shown that rat blood gene expression signatures are predictive of the toxic exposure to acetaminophen and can be used to monitor the exposure of the toxicant reflected in the human blood and in the rat liver. The study was based on a single hepatotoxicant which targeted a specific region of the rat liver (centrilobular), addressed discrimination of non\sub-toxic vs toxic (two class) and overdose exposure levels and demonstrated the superiority of gene expression markers over traditional clinical parameters in predicting the exposure. A more comprehensive analysis of compound-induced liver injury was performed *ab initio *using samples exposed to hepatotoxicants or compounds without known liver toxicity [9]. Expression profiles from 212 genes combined with a composite hepatotoxicity score were highly predictive of compound-induced liver injury. It would be useful to be able to identify gene expression patterns as a diagnostic signature for prediction of the (multiclass) level of necrosis as the general phenotypic response that is commonly manifested from toxic exposure to a compendium of stressors which targets various regions of the liver.

In our study, gene expression data from rat livers exposed to a compendium of hepatotoxicants (Lobenhofer et al., in press) was used to identify gene expression patterns as a diagnostic signature which predicts the level of necrosis of the liver with a high degree of accuracy. The eight chemical compounds in the compendium (1,2-dichlorobenzene, 1,4-dichlorobenzene, bromobenzene, monocrotaline, N-nitrosomorpholine, thioacetamide, galactosamine and diquat) elicit some or no hepatotoxicity in male rat liver samples at one or more of the three time points (with either a low, medium or high dose exposure). A form of necrosis was the major lesion observed in a region of the liver from the toxic doses of the chemicals. Analysis of the liver and blood gene expression data using SVM classifiers within each dose\time group separately for each tissue type revealed compound-specific separation of the samples exposed to the hepatotoxicant (Lobenhofer et al., in press). Interestingly, the blood data consistently performed better than the liver data in separating the samples into compound groups in the majority of the dose\time point groups. To investigate the ability of liver genomic markers to predict the level of necrosis manifested in the livers of the animals exposed to the hepatotoxicants, we utilized a Random Forest classifier with an out-of-bag classification error and variable importance estimation procedure to select gene predictors of three classes of the level of necrosis that were derived according to 1) the five severity scores of the injury 2) the differentially expressed genes from an ANOVA model and 3) the Gene Ontology biological processes enrichment shared by adjacent necrosis levels. From this strategy, gene expression profiles from 21 informative genes were identified as a diagnostic signature which achieved 90%, 80% and 60% prediction accuracies of the level of necrosis against independent test data derived from rats exposed to acetaminophen, carbon tetrachloride, and allyl alcohol, respectively. In addition, it was determined that inflammation follows programmed cell death in response to the hepatotoxicants and the cytotoxic effects of TNF-α as well as a transcriptional regulation by JUN and TP53, and apoptosis-related genes possibly lead to necrosis.

## Results

### Manifestation of Necrosis

We used a data set containing 8 hepatotoxicants (see Table 1) to identify genes related to necrosis level. Table 2 shows the distribution of the severity level of the animals in each compound studied. The necrosis severity included five levels: none, minimum, mild, moderate and marked. None represents no sign of necrosis, minimal represents less than 5% of hepatocytes show necrosis, mild represents 5%–25% of hepatocytes are necrotic, moderate means 26%–50% of hepatocytes show necrosis and marked means more than 50% of liver cells are necrotic.

**Table 1:** Experimental design of the training and test samples

Compound	Time (hr)	Dose (mg/kg body weight)			Observed Hepatotoxicity
			
Training		Low	Mid	High	
1,2-dichlorobenzene	6/24/48	15	150	1500	centrilobular necrosis
1,4-dichlorobenzene	6/24/48	15	150	1500	centrilobular necrosis
bromobenzene	6/24/48	25	75	250	centrilobular necrosis
diquat	6/24/48	5	10/20	25	centrilobular, midzonal, focal necrosis
galactosamine	6/24/48	25	100	400	multifocal necrosis
monocrotaline	6/24/48	10	50	300	centrilobular, midzonal necrosis
N-nitrosomorpholine	6/24/48	10	50	300	centrilobular necrosis
thioacetamide	6/24/48	15	50	150	centrilobular necrosis

Test					

acetaminophen (NCT 008)	6/24/48	50	150	1500/2000	
acetaminophen (NTP)	6/18/24/48	50	150	1500/2000	centrilobular necrosis
acetaminophen (informatics challenge)	3/6/12/24		150	1500/2500	
carbon tetrachloride	3/6/24/72	15	750	2000	centrilobular necrosis
Ally Alcohol	6/24/48/72	10	20	40/50	periportal necrosis

**Table 2:** Necrosis severity and distribution in each compound study of the training data

Necrosis Observation (% of hepatocytes showing necrosis)	No Sign	<5%	5%–25%	26%–50%	>50%	Sample size
Necrosis Level	0	1	2	3	4	
1,2-dichlorobenzene	17	8	5	2	2	34
1,4-dichlorobenzene	31	4	1	0	0	36
bromobenzene	16	7	5	0	8	36
diquat	50	10	6	4	2	72
galactosamine	18	7	8	2	1	36
monocrotaline	16	11	1	0	4	32
N-nitrosomorpholine	12	17	2	1	4	36
thioacetamide	4	18	1	6	7	36
Total sample size	164	82	29	15	28	318

### Gene Expression Changes Transition with Severity of Necrosis

Given the groups of samples according to the manifestation of hepatocyte necrosis, we extracted genes from the microarray data that have expression levels that are significantly different between severity levels. The severity levels of necrosis were transformed to indicator variables 0, 1, 2, 3 and 4 denoting none, minimal, mild, moderate and marked necrosis, respectively. An unbalanced one-way ANOVA was constructed with the level of necrosis as the only factor. To find significantly differentially expressed genes between two adjacent necrosis levels, four estimations of the comparison of necrosis score 0 vs. 1, 1 vs. 2, 2 vs. 3 and 3 vs. 4 were performed. Table 3 summarizes the number of differentially expressed genes between two adjacent necrosis scores under a series of Bonferroni multi-test correction thresholds. The number of significant differentially expressed genes for comparisons of necrosis score 1 vs. 2 and score 3 vs. 4 are much fewer than that for comparison of necrosis score 0 vs. 1 and score 2 vs. 3. This suggests that the expression of genes in the samples of the liver between minimal and mild necrosis and between moderate and marked necrosis are not much different. The bulk of the difference is between liver samples with no necrosis and those with minimal necrosis and between those with mild necrosis and with moderate necrosis.

**Table 3:** Number of DEGs between two adjacent necrosis levels after Bonferroni multi-test correction

Bonferroni p-value threshold	level 1 vs. 0	level 2 vs. 1	level 3 vs. 2	level 4 vs.3
0.0001	1592	14	336	31
0.001	1960	33	490	50
0.01	2404	90	695	91
0.05	2683	171	910	131

* A total of 20,500 genes from the Agilent platform were tested.

### Inflammation Follows Programmed Cell Death in Response to Hepatotoxicant Exposure

**Figure 1:**
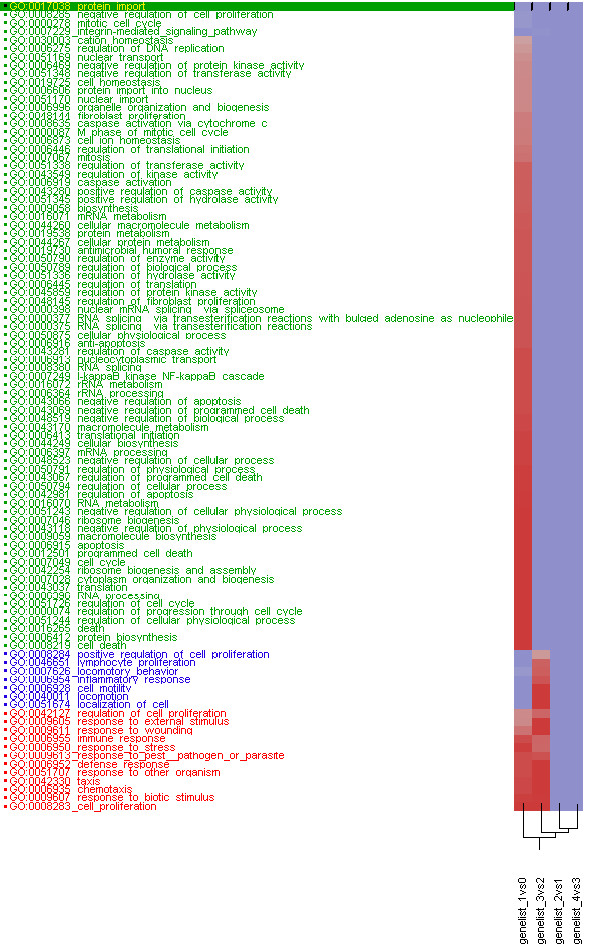
**High-Throughput GoMiner analysis of 4 groups of over-expressed genes.** There are 4 groups of significantly differentially expressed gene lists, one for comparison of necrosis level 0 vs. 1 with 2683 significant genes, one for comparison of necrosis level 1 vs. 2 with 171 significant genes, one for comparison of necrosis level 2 vs. 3 with 910 significant genes, and one for comparison of necrosis level 3 vs. 4 with 131 significant genes. The four gene lists are labeled on the bottom of the figure and over-represented biological processes are labeled at the left of the figure. The red color indicates that the p-value is smaller than the FDR rate of 0.05 whereas blue represents p-values larger than an FDR rate of 0.05. The smaller the p-value, the more intense the color.

Gene Ontology analysis was performed on four lists of significantly differentially expressed genes from the comparisons of two adjacent necrosis scores which met the Bonferroni 0.05 threshold. Figure 1 shows GoMiner analysis of over-expressed genes from the four lists of gene. The over-expressed genes in the comparison of necrosis score 1 vs. 2 and score 3 vs. 4 do not show any significant biological processes, while the over-expressed genes in the comparison of necrosis score 0 vs. 1 and 2 vs. 3 yielded several significant biological processes. One example is the biological process for inflammatory response. It is over-represented in the gene set discriminating necrosis level 2 and 3 but not when comparing level 0 and 1. The programmed cell death pathway is over-represented in this latter comparison. Therefore, it appears that after exposure of the liver to these hepatotoxicants in rats, programmed cell death is activated in the samples that exhibited mild or minimal necrosis while the inflammatory response is activated in the liver samples showing moderate or marked necrosis. These results, suggesting that inflammation follows programmed cell death, are in-line with recent finding that the mechanisms by which apoptosis promotes an inflammatory response may be related to death receptor-initiated signaling cascades [10,11].

Both the ANOVA statistical analysis and GoMiner biological processes analysis resulted in the conclusion that the gene expression, biological pathways and processes in the liver samples that manifest minimal or mild necrosis are very similar and difficult to differentiate from one another. The same is the case for liver samples revealing moderate or marked necrosis. However, the biological response in the liver of the samples with necrosis level comparisons of none vs. minimum and mild vs. moderate resulted in significant biological differences. Thus, for the purpose of this study, we combined the five necrosis severity scores into three necrosis levels, (no necrosis as level 0, minimal and mild necrosis as level 1, and moderate and marked necrosis as level 2) in order to identify genes that can predict the level of necrosis with a high degree of certainty from samples that share a common biological response. In the end, the sample size in each level increases and the statistical power to differentiate the newly defined levels of necrosis improves.

### Gene Classifiers that Predict Necrosis

#### Selection of Predictor Genes

First, a one-way ANOVA was fitted to extract the significant differentially expressed genes among the three newly defined necrosis levels. The same one-way ANOVA model as described above was applied with the modification that the necrosis level now was from 0 to 2 instead of from severity score 0 to 4. Three contrasts were performed to identify differentially expressed genes between level 0 vs. 1, 1 vs. 2 and 0 vs. 2. From this analysis, 8561 genes were significantly differentially expressed either in the contrast of level 0 vs. 1 or in the contrast of level 1 vs. 2 using Bonferroni multi-test correction at p-value threshold of 0.05 (See Additional file 1). Gene Ontology analysis performed on the two gene lists generated from the 0 vs. 1 and 1 vs. 2 level comparisons from the redefined groups reconfirms that inflammation follows programmed cell death when the samples are exposed to the hepatotoxicants (See Additional file 2 and See Additional file 3). Inflammation is significant only in the 1 vs. 2 comparison while program cell death is more significant in 0 vs. 1 comparison.

For building a model for prediction, all the normalized data from the 318 arrays (treated animals) were used as a training set. The three necrosis levels were used as the class labels and the 8561 genes as the predictors. Random Forest and GEMS-SVM were two classification approaches used for prediction.

#### Prediction with Random Forest

The Random Forest classification method selected 21 genes which have an out-of-bag (OOB) data error rate of 0.104 and standard deviation of 0.017. The names and annotation of the 21 selected genes are listed in Table 4. The list contains several genes related to inflammatory disease, cell-to-cell signaling and interaction, cell death, cellular movement, immune response, and cell organization.

**Table 4:** Annotation of the 21 selected Agilent probes using the Random Forest classification approach

Agilent Probe	Gene Acc. #	Gene Name	Description
A_42_P458530	NM_139342	Ripk3	Rattus norvegicus receptor-interacting serine-threonine kinase 3 (Ripk3)
A_42_P487811	AW914054	Sema4g_predicted	Sema domain, immunoglobulin domain (Ig), transmembrane domain (TM) and short cytoplasmic domain, (semaphorin) 4G (predicted)
A_42_P507284	NM_013111	Slc7a1	Rattus norvegicus solute carrier family 7 (cationic amino acid transporter, y+ system), member 1 (Slc7a1)
A_42_P517381	NM_133298	Gpnmb	Rattus norvegicus glycoprotein (transmembrane) nmb (Gpnmb)
A_42_P532103	NM_019905	Anxa2	Rattus norvegicus annexin A2 (Anxa2)
A_42_P594863	AI144754	Rnd1	Rho family GTPase 1 (predicted)
A_42_P621642	XM_341964	Lsp1	Rattus norvegicus similar to Lsp1 protein (LOC361680)
A_42_P695401	NM_031530	Ccl2, MCP-1	Rattus norvegicus chemokine (C-C motif) ligand 2 (Ccl2)
A_42_P710382	BF412297	TC466815	Transcribed locus
A_42_P730684	XM_214096	LOC289801	Rattus norvegicus similar to uridine phosphorylase (LOC289801)
A_42_P768467	BQ207775	RGD1305887	Similar to RIKEN cDNA 2310057H16 (predicted)
A_42_P809565	NM_139324	Ehd4	Rattus norvegicus EH-domain containing 4 (Ehd4)
A_43_P10621	AI177116	Vasp_predicted	Vasodilator-stimulated phosphoprotein (predicted)
A_43_P11353	BC083855	Lcp1	Lymphocyte cytosolic protein 1 (predicted)
A_43_P11621	NM_012924	Cd44	Rattus norvegicus CD44 antigen (Cd44)
A_43_P12519	NM_031114	S100a10	Rattus norvegicus S100 calcium binding protein A10 (calpactin) (S100a10), mRNA [NM_031114]
A_43_P12698	NM_031832	Lgals3	Rattus norvegicus lectin, galactose binding, soluble 3 (Lgals3)
A_43_P12940	NM_053812	Bak1	BCL2-antagonist/killer 1
A_43_P13182	NM_133416	Bcl2a1	B-cell leukemia/lymphoma 2 related protein A1
A_43_P14045	AW914054	Sema4g_predicted	Sema domain, immunoglobulin domain (Ig), transmembrane domain (TM) and short cytoplasmic domain, (semaphorin) 4G (predicted)
A_43_P15660	BC079312	Cxcl16	similar to chemokine (C-X-C motif) ligand 16

#### Prediction with GEMS

Classifiers used for prediction can be data dependent. Therefore, we also used the Gene Expression Model Selector (GEMS) approach for prediction. The best performing model selected by GEMS used a gene selection method consisting of a signal-to-noise ratio in a one-versus-rest fashion followed by a multicategory support vector machines method by Weston and Watkins [12]. Six genes were selected from the prediction model achieving a minimum cross validation error with prediction accuracy of 89.6% on the training data (Tables 5 and 6). Five of the six genes were also selected as predictors using the Random Forest approach except for inosine monophosphate (IMP) dehydrogenase 1. This gene catalyzes the rate-limiting reaction of *de novo *GTP biosynthesis at the inosine monophosphate metabolic branch point and therefore is involved in the regulation of cell proliferation. The prediction results on the training and test data from both classifiers are showed (See Additional file 4).

**Table 5:** Annotation of the 6 selected Agilent probes using GEMS-SVM

Agilent Probe	Gene Acc. #	Gene Name	Description
A_42_P458530 *	NM_139342	Ripk3	Rattus norvegicus receptor-interacting serine-threonine kinase 3 (Ripk3)
A_42_P507284 *	NM_013111	Slc7a1	Rattus norvegicus solute carrier family 7 (cationic amino acid transporter, y+ system), member 1 (Slc7a1)
A_42_P695401 *	NM_031530	Ccl2, MCP-1	Rattus norvegicus chemokine (C-C motif) ligand 2 (Ccl2)
A_42_P768467 *	BQ207775	RGD1305887	Similar to RIKEN cDNA 2310057H16 (predicted)
A_43_P11307	XM_342650	Impdh1_predicted	Rattus norvegicus similar to Impdh1 protein (LOC362329)
A_43_P11621 *	NM_012924	Cd44	Rattus norvegicus CD44 antigen (Cd44)

* Overlap with the 21 selected Agilent probes using Random Forest

**Table 6:** Prediction accuracy of the training and test data sets using Random Forest and GEMS-SVM

	Compounds	Sample size	Prediction Accuracy	
			
			Random Forest	GEMS
Training	1,2-dichlorobenzene	34	91.20%	85.30%
	1,4-dichlorobenzene	36	94.40%	94.40%
	bromobenzene	36	100%	94.40%
	diquat	72	86.10%	88.90%
	galactosamine	36	83.30%	86.10%
	monocrotaline	32	84.40%	84.40%
	N-nitrosomorpholine	36	94.40%	100%
	thioacetamide	36	86.10%	83.30%
	All training	318	89.60%	89.60%
Test	acetaminophen (NCT)	36	88.90%	88.90%
	acetaminophen (NTP)	64	87.50%	87.50%
	acetaminophen (Informatics Challenge)	108	90.70%	89.80%
	carbon tetrachloride	72	77.80%	77.80%
	Ally Alcohol	95	64.20%	59%

#### Comparison of Results Between Random Forest and GEMS

The same average prediction accuracy of 89.6% on the training data was achieved using the 21 genes selected by Random Forest and the 6 genes selected by the GEMS-SVM classifier (Table 6). Comparison of the prediction calls for the training data set from the two approaches indicates that the GEMS-SVM approach tends to misclassify samples towards the lower end of the necrosis level than the Random Forest approach (Table 7). Assessing the prediction accuracy for each compound in the training data set revealed that different compounds show different prediction accuracies, ranging from 83.3% to 100% (Table 6). The thioacetamide, monocrotaline, galactosamine, and diquat exposed samples always showed lower prediction accuracies (between 83%–89%) using both the Random Forest and GEMS-SVM approaches. The bromobenzene, 1,4-dichlorobenzene and N-nitrosomorpholine exposed samples were typically predicted with accuracies greater than 94% using either the Random Forest or GEMS-SVM approaches. The samples exposed to1,2-dichlorobenzene show better prediction accuracy using the Random Forest approach than the GEMS-SVM approach.

**Table 7:** Prediction accuracy for all the training data samples using Random Forest and GEMS-SVM

		Random Forest			GEMS		
			
		Predict			Predict		
		0	1	2	0	1	2
TRUE	0	151	12	1	155	9	0
	1	11	94	6	15	94	2
	2	0	3	40	0	7	36

**Figure 2:**
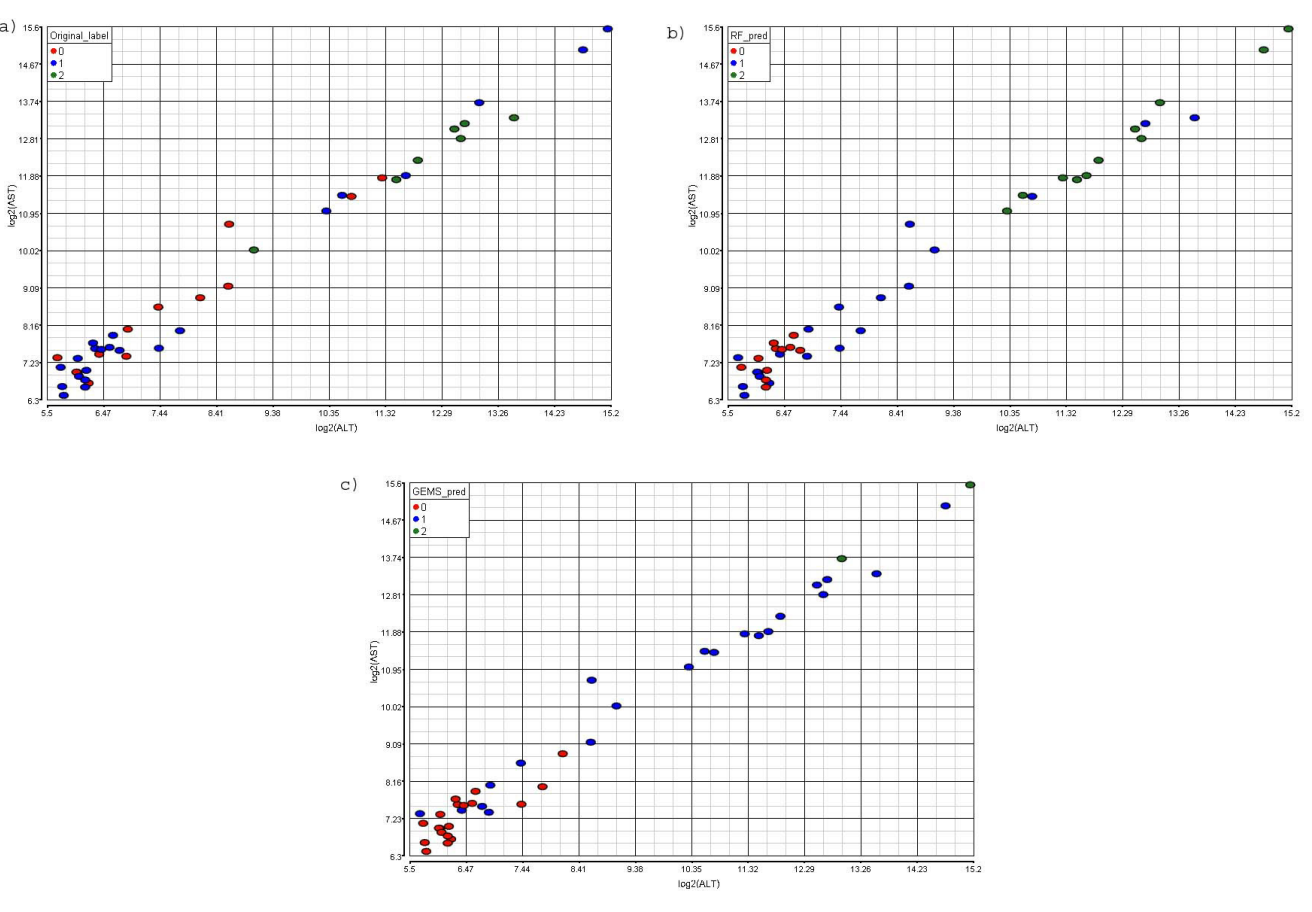
**The scatter plot of log2 (ALT) and log2 (AST) levels of all 42 disagreement animals from Random Forest and GEMS-SVM classifier colored by the class label.** (a) The scatter plot is colored by the necrosis class label according to the redefined groups; (b) The scatter plot is colored by the Random Forest predicted label; (c) The scatter plot is colored by the GEMS-SVM predicted label.

**Figure 3:**
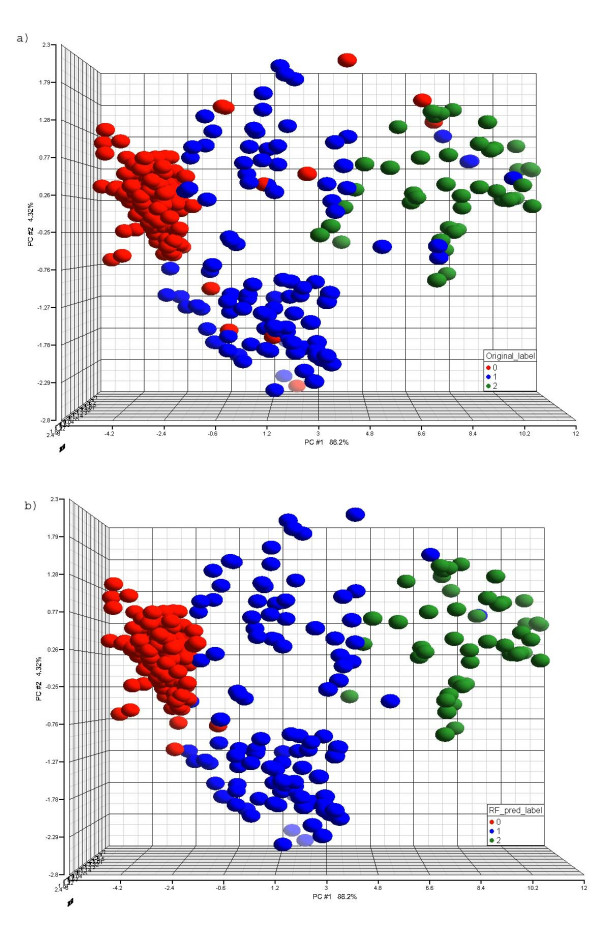
**PCA using the 21 selected genes.** The necrosis levels increase from left to right. The red color represents animals with necrosis label 0, blue represents animals with necrosis label 1 and green represents necrosis level 2. The color in (a) represents the original class label, while in (b) the color represents the Random Forest predicted class label.

Both the Random Forest classifier and GEMS-SVM classifier misclassified 33 animals from the training set. There are 24 animals in the overlap; thus, a total of 42 animals were misclassified either from the Random Forest approach or from the GEMS-SVM approach. Further verification using clinical chemistry data was performed for animals where there was disagreement between the predicted necrosis level and the necrosis class label according to the redefined groups (See Additional file 5). The correlation analysis (Table 8) and scatter plot (Figure 2) were performed on the data to identify the concordance of log_2_ALT/log_2_AST level, the predicted class label, and the necrosis class label based on the redefined groups. The Random Forest and GEMS-SVM predicted labels for the misclassified samples were more consistent with ALT and AST levels than the necrosis class label (Figure 2, Table 8). The Random Forest and GEMS-SVM predicted labels for the correctly classified samples were just as consistent (correlations > +0.83) with log_2_ALT and log_2_AST levels as the necrosis class label (Data not shown). This result corroborates our prediction call. Comparing the scatter plot colored by the Random Forest predicted label (Figure 2b) vs. colored by the GEMS-SVM predicted label (Figure 2c) on those misclassified samples revealed that the Random Forest method tends to predict the samples more towards a severe necrosis level while the GEMS-SVM approach tends to predict them towards a less severe necrosis level (see Table 7 as well). Figure 3 is a principal component analysis (PCA) on all 318 training animals using the 21 selected genes illustrating the level of necrosis separated along the PC1. Figure 3a is colored by the necrosis class label and Figure 3b is colored by the predicted label. Both figures show that the level of necrosis increases from left to right along the PC1.

**Table 8:** Correlation analysis of ALT and AST with the necrosis class, Random Forest predicted or GEMS-SVM predicted label

	Necrosis class label	GEMS_pred	RF_pred	Log_2_ALT	Log_2_AST
Necrosis class label	1				
GEMS_pred	0.095585	1			
RF_pred	0.146186	0.680875	1		
Log_2_ALT	**0.448879**	**0.749506**	**0.758779**	1	
Log_2_AST	**0.417822**	**0.778307**	**0.743858**	0.990228	1

To validate the prediction using the selected genes, the classifier was applied to the test data. The chemical compounds in the testing data sets (see Table 1) include three different studies of rat livers exposed to acetaminophen, one to carbon tetrachloride and one to allyl alcohol. When the Random Forest classifier was applied to the acetaminophen-treated sample data, the prediction accuracies were 88.9%, 87.5% and 90.7% respectively for the data sets from the National Center for Toxicogenomics study #8 (NCT008), National Toxicology Program (NTP) and National Center for Toxicogenomics informatics challenge study (NCT informatics challenge). The Random Forest classifier produced 77.8% prediction accuracy for the carbon tetrachloride-treated test samples. Surprisingly, only 64.2% prediction accuracy was achieved with the classifier applied to the allyl alcohol-treated test samples (Table 6). Similarly, the GEMS-SVM classifier with the 6 predictive genes achieved prediction accuracies of 88.9%, 87.5% and 89.8% respectively for three acetaminophen data sets from NCT008, NTP and NCT informatics challenge. In the case of the carbon tetrachloride test samples, the GEMS-SVM classifier achieved the same prediction accuracy (77.8%) as the Random Forest classifier. Not surprisingly, similar to the Random Forest prediction of the test data, the GEMS-SVM classifier model performed poorly on the allyl alcohol sample set achieving only 59% prediction accuracy. Generally speaking, the GEMS-SVM classifier model performed slightly worse than the Random Forest model on the test data, probably due to the smaller number of genes in the former model.

To test the significance of the 21 genes in the classifier 10,000 samples were generated by randomly selecting 21 genes from the array to use for training of the Random Forest classifier and testing on the test data. The median prediction accuracy for the 10,000 randomly generated samples is 70%. About 4.7% of the random samples achieved a prediction accuracy greater than 76%. Only 4 out of the 10,000 samples achieved prediction accuracy greater than or equal to 0.81 (accuracy for the 21 genes using the Random Forest classifier). Therefore, the significance of the prediction accuracy of the selected 21 genes in the Random Forest classifier has a p-value < 0.0005.

### Biological Pathway and Gene Network Analyses

**Figure 4:**
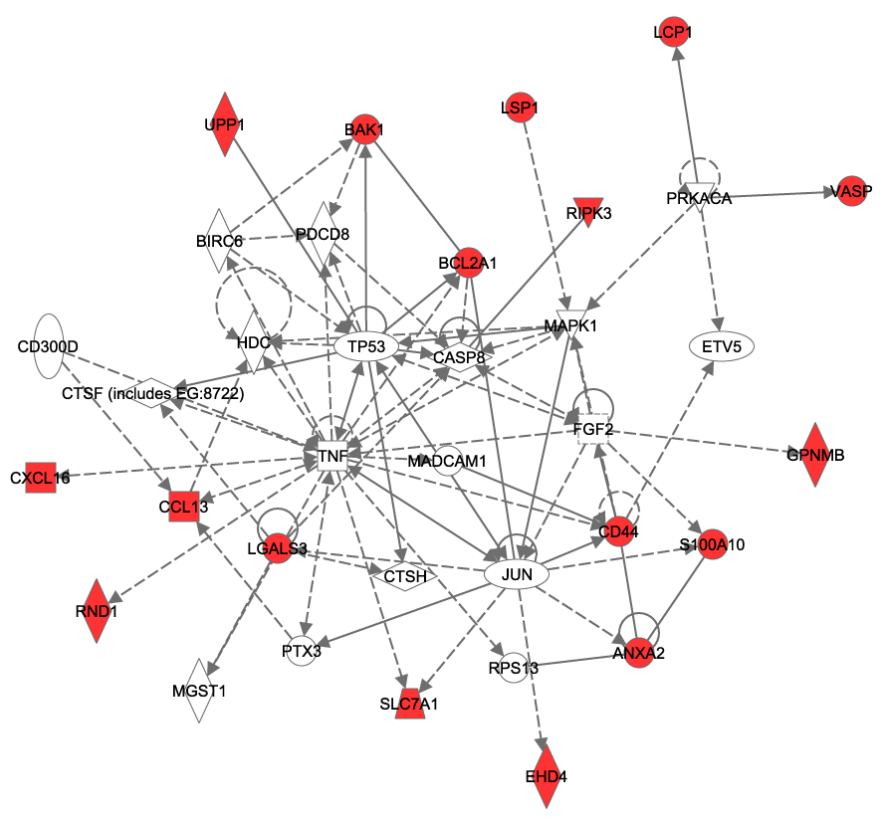
**Pathway analysis.** The 21 gene probes corresponding to 20 genes, were analyzed by Ingenuity Pathway Analysis software version 5.1. Eighteen out of 20 genes were annotated for gene network construction. Seventeen out of 20 over-expressed genes were mapped to the same network space. The red nodes represent the 17 selected genes. The pathway analysis revealed a central regulating role of tumor necrosis factor (TNF), Jun and TP53.

**Figure 5:**
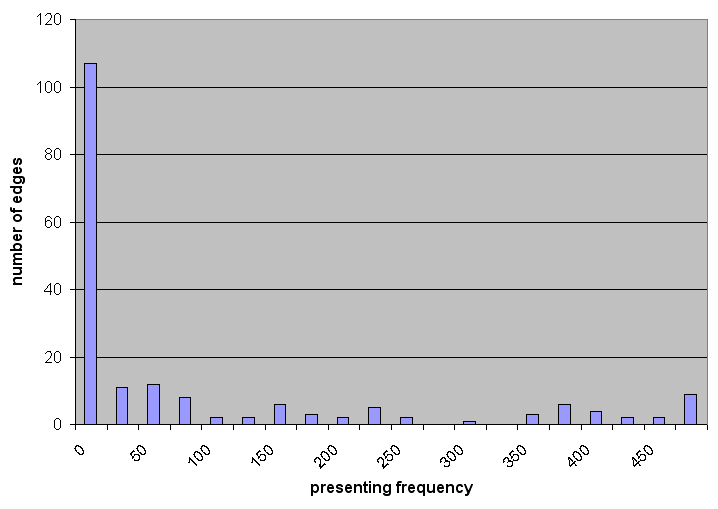
**Histogram of the number of edges reoccurring in 500 networks.** The 187 possible edges present in at least one of 500 networks are plotted. The 107 out of 187 edges are presented less than 25 times in 500 networks. The x-axis is the number of occurrences of the edges and the y-axis is the frequency.

**Figure 6:**
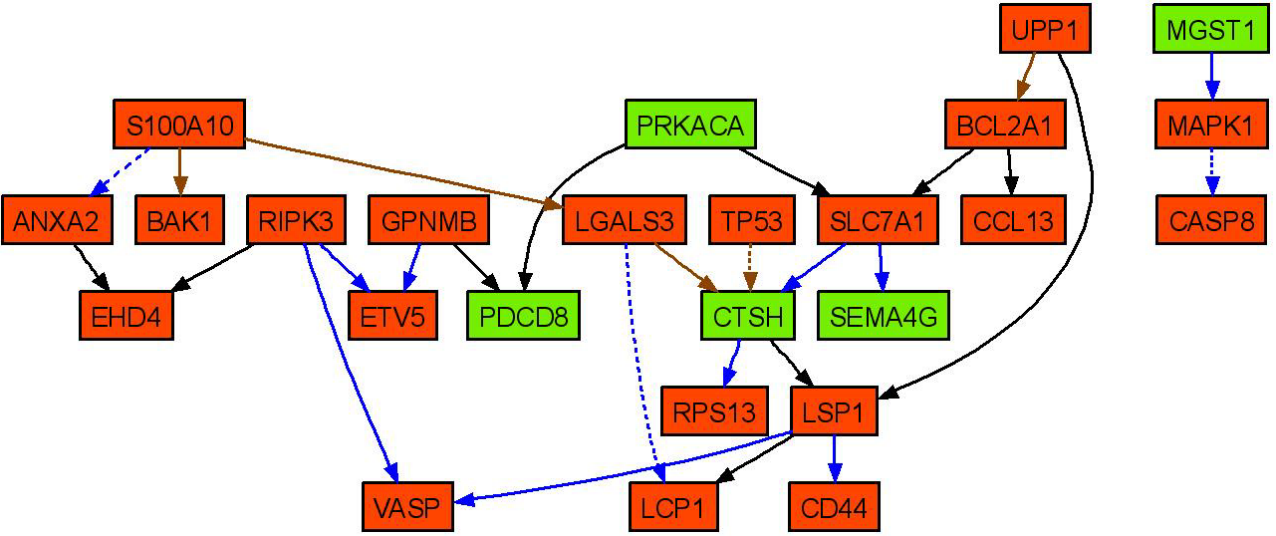
**Reconstructed (consensus) gene network.** The reconstructed Bayesian network was generated from the gene expression data from a total of 32 gene profiles. It depicts the statistical dependence between the transcript levels of the genes. The red nodes represent the up-regulated genes and green nodes represent the down-regulated genes both when the necrosis severity increases. Blue edges have a probability greater than 0.9, brown edges between 0.8 to 0.9, and black edges between 0.6 to 0.8. The dashed lines represent the edges consistent with the Ingenuity Pathway Analysis in Figure 4.

Pathway analysis of the 21 predictor genes revealed a central regulating role of tumor necrosis factor (TNF), Jun and TP53 (Figure 4). The majority of the predictor genes (17 out of 21) are regulated in their expression by those transcription factors. Bayesian gene networks were reconstructed using the discretized expression profile of all mapped genes from the training samples. The edges between the genes denote inferred interactions. Animals treated with a low dose (i.e. non-toxic dose) of the agents don't usually manifest necrosis. Only samples treated with a high dose (i.e. the more toxic dose) of the agents were selected (including the samples from the training data set excluding 1,4-dichlorobenzene) for reconstruction of the Bayesian networks. The networks with the highest posterior probability of the model given the data were collected from a large number of heuristic searches. The frequency distribution histogram (Figure 5) details the number of edges that reoccur in the 500 networks that were reconstructed. There are potentially 1024 edges for the 32 genes used to generate the networks (including the self-directed edge), 837 edges not presented (found zero times), 17 edges presented over 400 times, and 10 edges presented between 300 and 400 times. The probability of each edge being presented was calculated using weighted average scoring. Figure 6 shows the consensus network recreated using the highest scoring network from each of the 500 runs of the algorithm, including all edges with a probability of being presented greater than 0.6. Four gene-to-gene interactions (S100 Calpactin [S100a10] with annexin A2 [Anxa2], Lectin, galactose binding, soluble 3 [Lgals3] with Epidermal Langerhans cell protein [Lcp1], Tumor protein p53 [TP53] with Cathepsin H [Ctsh] and Mitogen activated protein kinase 1 [Mapk1] with Caspase 8 [Casp8]) with probabilities greater than 0.8 are consistent with the interactions in the biological pathway generated from curation of scientific literature (Figure 4).

## Discussion

Hepatotoxicants particularly target the liver and cause a variety of liver injuries. One type of damage is necrosis, a degenerative process leading to cell death. We analyzed a compendium of gene expression data (see Lobenhofer et al., in press) acquired from rat livers exposed to hepatotoxicants in an attempt to define gene expression patterns as a signatures that are highly predictive of the level of necrosis. We used necrosis as a phenotypic anchor to identify genes which predict the level of necrosis of the rat liver with a high degree of accuracy.

A prevailing advantage of our study was identifying genes related to necrosis which may be directly related to cell death caused by exposure to the compounds while filtering out genes related to other mechanisms related to a single compound. This was accomplished by using the histopathologic class label of the biological samples for necrosis as defined by board certified pathologists. We then leveraged ANOVA pairwise contrasts of the severity groups and GoMiner analysis of the gene expression data to subset the samples into groups with similar phenotypic changes exhibited by necrosis of the liver and comparable overrepresentation of biological processes (Figure 1). Using these groups as class labels for the level of necrosis in the training set and two gene selectionification approaches (Random Forest and GEMS-SVM), we were able to identify subsets of genes which yielded a low prediction error rate during cross validation of the classifiers. Preliminary analysis of the liver gene expression data within each dose\time group by Lobenhofer et al. (in press) revealed compound-specific separation of the samples. Although classification of the blood data was better in higher dose groups at the later time points as compared to the liver data, classifiers derived from it was not able to predict animals in some cases where the hepatotoxicant elicited a different phenotypic response with the animals of a particular dose\time group. Interestingly, concordance analysis of ALT and AST enzyme levels with the class label of the level of necrosis and our predicted class labels revealed that the latter is more consistent with the enzyme levels. One potential reason for this could be that the microarray samples are from the whole liver whereas the histopathology samples used for scoring the extent of necrosis are only from two independent slices of the liver specimen. In a limited study, Heinloth et al. [13] also showed that gene expression analysis is more informative than histopathologic evaluations and offers unique advantages to liver biopsy evaluations. Another explanation could be that certain animals may develop the phenotype at a later time point after treatment than the time point when the samples were taken for analysis. Therefore, the histopathology samples may not completely represent the liver toxicity. However, our gene expression analysis, redefining of the class labels for the level of necrosis exhibited in the samples and selection of predictor genes for necrosis are geared towards capturing the biological processes and mechanistic pathways that may govern the manifestation of the phenotype from a low level of necrosis to its highest level.

Using independent gene expression data sets acquired from the exposure of rat liver samples to a different set of hepatotoxicants, we show that the prediction accuracies of either of the two classifiers were roughly 80% overall but approximately 90%, about 80% and around 60% for acetaminophen-, carbon tetrachloride- and allyl alcohol-exposed samples, respectively with a p-value < 0.0005 for the significance of the prediction using the Random Forest classifier (Table 6). The dramatic difference in prediction accuracy could be related to bioactivation mechanisms involved in the manifestation of centrilobular necrosis in the case of acetaminophen- and carbon tetrachloride-toxicity as opposed to periportal necrosis in the case of allyl alcohol-toxicity. In the former, the abundance of cytochrome P450 plays a critical role whereas in the latter, higher oxygen levels are responsible [2]. Another reason may be a site-specific batch effect since the allyl alcohol microarray data was generated at a different location than the acetaminophen and carbon tetrachloride microarray data. The training and testing samples are plotted together using PCA of the expression data from the signature of the 21 selected genes (See Additional file 6). The testing samples show similar distributions as the training samples and also indicate that the necrosis level increases from right to left along PC1. The acetaminophen- and carbon tetrachloride – exposed samples show a similar data dispersion range as the training samples while the ally alcohol – exposed samples are more compressed along the first PC.

Genes from our predictive models (See Tables 4 and 5) have biological functions related to the regulation of apoptosis (Ripk3 and Bcl2a1) or are involved in a chemokine\inflammatory response (Ccl13 also known as CCL2/MCP-1), Cxcl16 and Lgals3. Pathway analysis of the predictor genes revealed a central regulating role of tumor necrosis factor (TNF), Jun and TP53 (Figure 4). The majority of the predictor genes in the signature (17 out of 21) are regulated in their expression by these transcription factors. Therefore, our results are generally in agreement with the current hypothesis that TNF mediates liver injury and genes such as Jun and TP53 are closely involved in necrotic changes in response to exposure to some hepatotoxicants [14-21]. Surprisingly, monocyte chemoattractant protein-1 (MCP-1), a serum factor gene and chemokine that is in our predictor gene list, was shown to have its protein product differentially expressed in acetaminophen-treated rats [22] and is induced by TNF-α [23]. This regulation might be a reflection of a repair process following liver injury by acetaminophen-toxic exposure or could be a contributor to the insult. Although the role of MCP-1 in liver injury is controversial [22], new evidence using MCP-1 deficient mice suggests that interference of the gene's expression is sufficient for altering the processes that lead to severe carbon tetrachloride-induced liver injury [24]. However, caution must be taken as a more complicated biological response to liver injury is likely since there are hepatotoxicants, such as monocrotaline – (MCT, a pyrrolizidine alkaloid plant toxin), where an inflammatory response ensues secondarily to injury of the liver and TNF-α appears to not be primarily responsible for the hepatotoxicity [25]. In addition, transcription factors such as TNF-α and TP53 have both pro- and anti-apoptotic effects. TP53 keeps the cell from progressing through the cell cycle if there is damage to DNA but can also cause the cell to enter apoptosis if the damage cannot be repaired. Similarly, TNF-α can induce pro-apoptotic signaling mechanisms [26] or induce resistance against apoptosis [27] depending on the overall condition of the cell and its microenvironment.

The reconstructed Bayesian network from the toxic exposures of the hepatotoxicants (Figure 6) revealed several gene interactions that are consistent with interactions in the pathway that was generated from curated scientific literature (Figure 4) and points to apoptosis-related genes in necrosis-mediated toxicity. Bear in mind that the network is a consensus one, has only positive, one-way, acyclic interactions and was generated from microarray data alone using a limited number of genes. However, the confidence of each gene-to-gene edge (interaction) was calculated by performing 500 simulated annealing searches.

## Conclusion

We have demonstrated that gene expression signatures can predict, with a high degree of accuracy, the severity of necrosis of the liver elicited by acute exposure of rats to a variety of hepatotoxicants. First, the class discovery approach using ANOVA and GoMiner pathway analysis provided well-defined groups. This step is important since the redefined groups are more correlated with the liver injury as measured by ALT and AST enzyme levels. In addition, the gene selection strategy using Random Forest and GEMS-SVM improved the accuracy of predicting the severity of necrosis. Furthermore, the gene expression signature led to the identification of the molecular pathways that exhibited biological relevance to the manifestation of necrosis. Finally, pathway and gene network analyses revealed several gene interactions suggesting that apoptosis may be a consequence of the chain of events stemming from drug-induced liver injury and it leads to, or is concomitant with, the manifestation of necrosis of the liver from exposure of the hepatotoxicants in rats.

## Methods

### Experimental Design

The training data set is comprised of studies from the exposure of rats to one of eight compounds (1,2-dichlorobenzene, 1,4-dichlorobenzene, bromobenzene, monocrotaline, N-nitrosomorpholine, thioacetamide, galactosamine and diquat). All eight compounds were studied using standardized procedures, i.e. a common array platform, experimental procedures and data retrieving and analysis processes [28] (Lobenhofer et al., in press). For each compound, four to six male, 12 week old F344 rats were exposed to a low dose, mid dose(s) and a high dose of the toxicant and sacrificed at 6, 24 and 48 hr later (Table 1). At necropsy, liver and blood were harvested for RNA extraction, histopathology, clinical chemistry and hematology assessments. For liver RNA, left liver lobes were flash frozen, pulverized, and RNA was extracted from a portion of the powder with the QIAGEN RNeasy Maxi Kits (QIAGEN, Valencia, CA). The test data is comprised of three compound data sets (acetaminophen, carbon tetrachloride, and ally alcohol). Acetaminophen data sets were collected from three different independent studies (NCT008, NTP and NCT informatics challenges). Studies used in the test data are not from the standardized procedures. For both the training and test samples, a time-matched vehicle control pool was made for each compound and each tissue by pooling equal amounts of RNA from each of the four control animals. Each treated animal was hybridized against a time matched control pool to the Agilent Rat Oligonucleotide Microarray (Agilent #G4130A) with a dye-swap technical replicate. Fluorescence intensities were measured with an Agilent DNA Microarray Scanner (Agilent G2565AA) and processed with the Agilent G2565AA Feature Extraction software.

### Normalization of the Microarray Data

The log_10_-ratio intensity value for each gene feature on the array was retrieved from the raw file of each array. Each array was normalized by subtracting the sample-median value. Then the dye-swap arrays from the same biological replicate were merged by averaging. After dye-swap merging, there were a total of 318 arrays, one for each treated animal.

### Histopathology

From the left liver lobes, two sections were taken and fixed in 10% formalin. After dehydration with ethanol, the liver sections were embedded in paraffin and H&E stained slides were made. These slides were evaluated by two independent pathologists and disagreements were resolved by a pathology working group review [29]. Hepatocyte necrosis was one of the observed lesions. The severity of necrosis was graded into 5 levels (none, minimal, mild, moderate and marked) by pathologists according to the percentage of hepatocytes that show necrosis (Table 2). The necrosis observation severity levels were then used as a class label for the samples in the training and test data sets.

### Clinical Chemistry

At sacrifice, blood was collected into serum separation tubes (BD Microtainer^® ^Tubes, BD, Franklin Lakes, NJ) and serum was separated. Clinical chemistry analyses (albumin, cholesterol, creatinine, direct bilirubin, total bilirubin, total bile acid concentrations, triglycerides, and activities of alanine aminotransferase [ALT], alkaline phosphatase, aspartate aminotransferase [AST], lactate dehydrogenase [LDH] and sorbitol dehydrogenase [SDH]) were performed on all rats at study termination. Serum levels of the established liver injury markers ALT and AST increase when the liver shows inflammation or hepatotoxicity.

### One-way ANOVA

To identify the genes that are significantly differentially expressed among the different levels of necrosis, an unbalanced one-way ANOVA was fitted for each gene,

*Y*_*ij *_= *μ *+ *N*_*i *_+ *ε*_*ij*_, where

*i *= 0...m (level of necrosis)

*j *= 1...*n*_*i*_, *n*_*i *_is the number of rats in necrosis level i.

*Y*_*ij*_, the gene expression value at the *j*^*th *^rat of *i*^*th *^necrosis level

μ, the mean of the expression of the gene for all rats from all necrosis levels

*N*_*i*_, the necrosis effect of the gene at level *i*

*ε*_*ij*_, the expression deviation of the gene at *j*^*th *^rat from the necrosis effect of level *i*

The number of rats in the different necrosis levels is different, leading to an unbalanced design with different numbers of replicates in each level of the necrosis factor. The significantly differentially expressed genes between two adjacent necrosis levels were identified by estimation statements. Bonferroni multi-test corrections are applied.

### Biological Processes Analyses

The gene symbols for all genes features on the Agilent chip were retrieved from the Stanford Genomics Resources S.O.U.R.C.E. database and used as the input of High-Throughput GoMiner [30]. GoMiner is used to test if selected genes in a gene list are over-represented for a particular biological process. Here, the total genes are all genes from the chip and the selected genes are the ones identified as significantly differentially expressed. The over-expressed genes and under-expressed genes of the selected differentially expressed genes were tested separately. For each gene list, every biological process was assessed for significance based on the Fisher's exact test and a p-value assigned based on the hypergeometric probability distribution. All p-values from each combination of a biological process and gene list were filtered using a false discovery rate (FDR) at 0.05, clustered and visualized using JAVA Treeview [31].

### Gene Selection and Classification Methods

#### Random Forest

The Random Forest classification approach grows many single classification trees and chooses the most popular vote over all trees in the forest [32]. Each tree uses the randomly selected samples (with replacement) as the training set. About one-third of the cases are left out of the selected samples, which is called the out-of-bag (OOB) data. The OOB data is used as the testing data to get an unbiased estimate of the classification error and to estimate variable importance. Two R packages, randomForest and varSelRf were used in the Random Forest classification.

#### GEMS

Gene Expression Model Selector provides several SVM-based binary or multi-category classification methods and several gene selection methods [33]. The software constructs and estimates models from all combinations of gene selection methods and classification methods, then reports the models with the minimum cross-validation error. This is a filtering approach to rank genes by the selection method and then include them (step by step from the top ranked one to the bottom ranked one) into the model. Ten-fold nested cross-validation was used to provide an unbiased estimation of the model performance. A linear polynomial kernel was selected for the SVM.

### Gene Network Reconstruction

Genes identified as significantly differentially expressed and then selected as a predictor from the Random Forest classifier were used in the Ingenuity Pathway Analysis (IPA) software (Ingenuity Systems, Redwood City, CA) to identify biological pathways that the genes are a component of. All genes within the network space from the pathway analysis were collected, their Agilent probes mapped and expression profiles retrieved. Thus, a total of 32 gene profiles were used for gene network reconstruction. Bayesian Network Inference with Java Objects (BANJO) developed by Dr. Alexander Hartemink was used to build Bayesian networks from the data [34]. The 32 gene profiles across 318 arrays were discretized into three levels using the prior frequencies of each class. Only 84 samples from the high dose treatments of the hepatotoxicants (except for 1,4-dichlorobenzene) were used for the network reconstruction. In each run, a heuristic algorithm based on simulated annealing searches for the network\model with the highest posterior probability of being generated from the data. We ran the algorithm 500 times and gathered the highest scoring network from each. The probability of the edges being presented was computed using weighted average scoring from all the models [35]. If an edge is presented in all 500 runs, the probability of it is exactly 1.

## Abbreviations

ALT: Alanine aminotransferase; AST: Aspartate aminotransferase; SDH: Sorbitol dehydrogenase; LDH: Lactate dehydrogenase; DEGs: differential expressed genes; OOB: out-of-bag; GEMS: Gene Expression Model Selector; RF: Random Forest; NCT: National Center for Toxicogenomics; NTP: National Toxicology Program; TNF: tumor necrosis factor; MCP-1: Monocyte chemoattractant protein-1; IPA: Ingenuity Pathway Analysis; BANJO: Bayesian Network Inference with Java Objects.

## Authors' contributions

LH conceived of the concept of the research and methodology for analysis, performed the analysis and wrote part of the manuscript. PRB provided the valuable suggestions for the concept of the research, for all aspects of the methodology and analysis of the data and also wrote part of the manuscript. ANH and RSP offered helpful suggestions for the experimental design of the studies and for the pathway analysis. Z–BZ offered helpful suggestions with the statistical analysis and the gene network reconstruction. All authors read and approved the final manuscript.

## Supplementary Material

Additional file 1 **8561 DEGs selected using ANOVA**. Table_S1.txt is a tab-delimited text file to be opened and viewed with any standard spreadsheet software. Click here for file

Additional file 2 **High-Throughput GoMiner analysis of 2 lists of over-expressed genes after redefining the groups**. Supplemental_Figure_1: There are 2 significant differentially expressed gene lists, one for comparison of necrosis level 0 vs. 1 with 3623 significant genes and the other for comparison of necrosis level 1 vs. 2 with 7436 significant genes. The two gene lists are labeled on the top of the figure and over-represented biological processes are labeled at the right of the figure. The red color indicates that the p-value is smaller than the FDR rate of 0.05 whereas black represents p-values larger than an FDR rate of 0.05. The smaller the p-value, the more intense the color. Click here for file

Additional file 3 **p-value of Gene Ontology analysis for the redefined necrosis groups**. Table_S2.txt is a tab-delimited text file to be opened and viewed with any standard spreadsheet software. Click here for file

Additional file 4 **Necrosis class label and prediction results from Random Forest and GEMS-SVM classifiers for all training and test samples**. Table_S3.txt is a tab-delimited text file to be opened and viewed with any standard spreadsheet software. Click here for file

Additional file 5 **Clinical chemistry and necrosis score for disagreement animals**. Table_S4.txt is a tab-delimited text file to be opened and viewed with any standard spreadsheet software. Click here for file

Additional file 6 **PCA plots of the training and test data together using the 21 selected genes**. Supplemental_Figure_2: Red color represents the training data and the blue color represents the test data. The circles represent samples with necrosis level 0, the triangles represent samples with necrosis level 1 and the pluses represent samples with necrosis level 2. (a) PCA plot of the training data with the three acetaminophen test data sets. The first three components explain 92.8% of the variability in the data; (b) PCA plot of the training data with the carbon tetrachloride test data. About 94.1% of the variability in the data is explained by the three components; (c) PCA plot of the training data with the ally alcohol test data. 94.3% of the variability is explained by the first three components. Click here for file
