# Supplementary figures and images for: Genes related to apoptosis predict necrosis of the liver as a phenotype observed in rats exposed to a compendium of hepatotoxicants
Source: BMC Genomics. 2008 Jun 16;9:288. doi: 10.1186/1471-2164-9-288 (PMC2478688; doi:10.1186/1471-2164-9-288)

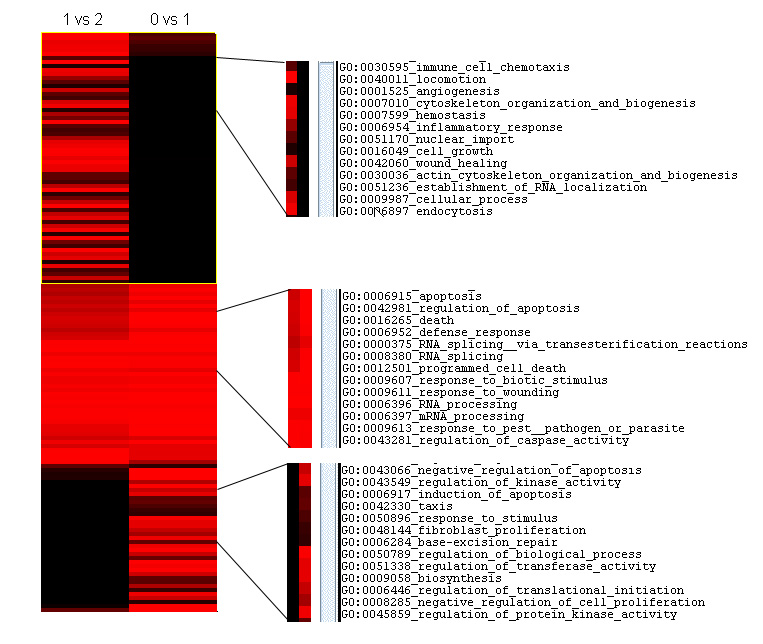

Supplement: Additional file 2 — High-Throughput GoMiner analysis of 2 lists of over-expressed genes after redefining the groups. Supplemental_Figure_1: There are 2 significant differentially expressed gene lists, one for comparison of necrosis level 0 vs. 1 with 3623 significant genes and the other for comparison of necrosis level 1 vs. 2 with 7436 significant genes. The two gene lists are labeled on the top of the figure and over-represented biological processes are labeled at the right of the figure. The red color indicates that the p-value is smaller than the FDR rate of 0.05 whereas black represents p-values larger than an FDR rate of 0.05. The smaller the p-value, the more intense the color. [file 1471-2164-9-288-S2.jpeg]

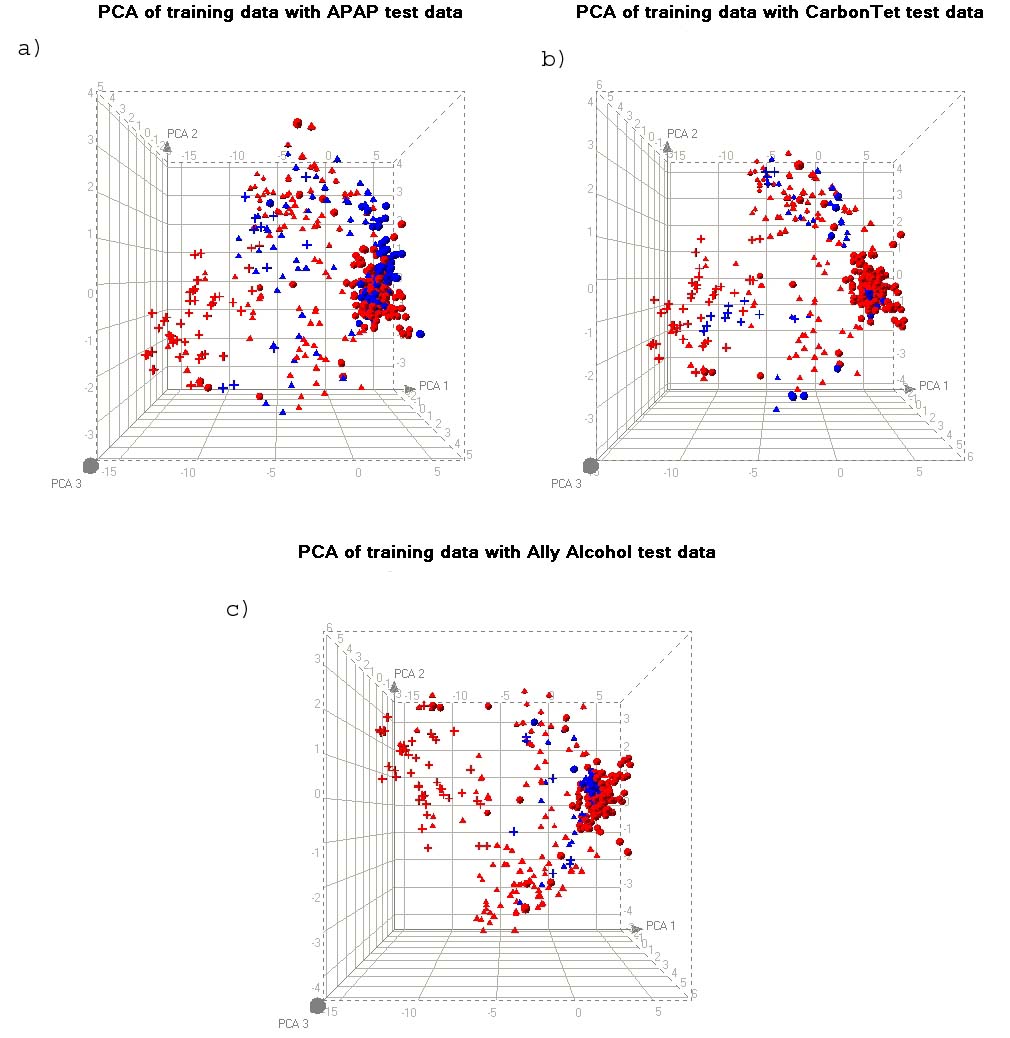

Supplement: Additional file 6 — PCA plots of the training and test data together using the 21 selected genes. Supplemental_Figure_2: Red color represents the training data and the blue color represents the test data. The circles represent samples with necrosis level 0, the triangles represent samples with necrosis level 1 and the pluses represent samples with necrosis level 2. (a) PCA plot of the training data with the three acetaminophen test data sets. The first three components explain 92.8% of the variability in the data; (b) PCA plot of the training data with the carbon tetrachloride test data. About 94.1% of the variability in the data is explained by the three components; (c) PCA plot of the training data with the ally alcohol test data. 94.3% of the variability is explained by the first three components. [file 1471-2164-9-288-S6.jpeg]
